# UAE university students’ experiences of virtual classroom learning during Covid 19

**DOI:** 10.1186/s40561-023-00225-1

**Published:** 2023-01-12

**Authors:** Monjurul Islam, Nurul Hijja Mazlan, Ghadah Al Murshidi, Mohammed Shamsul Hoque, S. V. Karthiga, Mohoshin Reza

**Affiliations:** 1 School of Language, Literacies & Translation, Universiti Sains Malaysia, Penang, Malaysia; 2 College of Computing, Informatics, and Media, Universiti Teknologi MARA, Shah Alam, Malaysia; 3 United Arab Emirates University, College of education, Al Ain, UAE; 4 Department of English, Daffodil International University, Dhaka, Bangladesh; 5 College of Science and Humanities, SRM Institute of Science and Technology, Kattankulathur, Chennai, India; 6 Department of English, Bangladesh University of Professionals, Dhaka, Bangladesh

**Keywords:** Virtual classroom (VC), Higher education, Survey, online, quantitative method

## Abstract

Virtual Classroom (VC) learning approaches have recently drawn considerable attention because they have the potential to encourage student engagement to ensure active and collaborative learning. Although research on online learning has gained visibility in recent times, VC learning has not received notable attention, especially in Gulf countries like the United Arab Emirates (UAE). The study examines students’ perception and experience of VC in a university in UAE during the COVID-19 pandemic in terms of its necessity and helpfulness. This research also examines the situational pressure of VC and aims to explain the reasons for its desirability and inevitability. As a current learning space beyond the conventional face-to-face classroom learning, VC learning is available in various forms and quality depending on creating opportunities for the learners’ participation. However, there are issues with VC practice too. Our analysis of survey data (*N* = 334) leads to portraying autonomous learning freedom in different learning environments in VC. We argue that students may resort to VC not because of its proven effectiveness but because of the necessity to continue addressing their learning needs. This study contributes to the general understanding of the online and traditional in-person classroom learning and virtual learning resources in the teaching of English as a globally desired language.

## Introduction

There are many ways in which virtual learning can be described as it covers a wide scope of online learning. “Virtual learning uses computer software, the Internet or both to deliver instruction to students. This minimizes or eliminates the need for teachers and students to share a classroom” (Van Beek, 2011, Para 3). This, therefore, became not only an option, but also the only solution for the continuation of teaching and learning through the Covid 19 pandemic within the UAE. Virtual learning can be 100% online, or it can be utilized to complement and enhance face to face teaching in methods such as Flipped Classroom (FC) or Blended Learning (BL). The Covid 19 pandemic saw all these variations being used to some extent within the UAE. The switch over was immediate and teachers and students had little or, no time to acclimatize to this new situation. It was the only way forward to allow students to continue learning during the lockdown period. Many studies (Ali, 2019; Farkhani et al., 2022; Sage et al., 2021; Sprenger & Schwaninger, 2021; Yaakub, 2020) have shown that virtual learning is the way to partake in online education amidst the hypercharge and great challenges in the world today for countries to thrive, transform, and ensure sustainability. It has been an officially recognized platform for filling in the gaps of traditional classroom teaching–learning approaches and it has rightly argued that VC has blurred the boundaries of face-to-face classroom teaching. It has become a critical component of the emerging global education industry (Hamid, et al., 2018).

Although research on different types of online learning is visible in recent times, the Virtual Classroom (VC), one of categories of EdTech (Education Technology) teaching and learning, is yet to receive notable attention particularly in Gulf countries like UAE (Ali, 2019; Yaakub, 2020). Researching VC is imperative in the age of globalization and the global spread of online education when proficiency of EdTech skills is linked to individual mobility and personal development. Policy recognition of this role of technology supported education is demonstrated by more and earlier introduction of online education in the curriculum in different parts of the world. Moreover, after post-pandemic teaching and learning, as we return to campus with improved EdTech skills, the challenge facing us is finding a new and effective balance between online instruction and face-to-face teaching. From the standpoint of this background, this study examines students’ experiences and understanding of VC in the UAE universities, a high-income country of 10.08 people in the Gulf region where the social desire of EdTech skills has accelerated the growth of VC learning, raised educational policy and planning challenges in a modern era. These questions implicate EdTech teaching and learning in general and VC learning, is used, in particular, as a crucial factor of the recent educational trends.

## Research in VC learning

Over the last two decades or so, VC learning has expanded around the globe (e.g., Keengwe & Kidd, 2010; Kemp & Grieve, 2014; Manegre & Sabiri, 2022; Palvia et al., 2018; Sage et al., 2021) and the COVID-19 situation has considerably accelerated educational institutions’ implementation of VC classes. Researchers have identified various paybacks for VC classes and one of the most significant benefits is its flexibility that allowing students make decisions about when and what they learn (Keengwe & Kidd, 2010; Kemp & Grieve, 2014). Moreover, recent studies have shown that VC learning is pedagogically promising because it motivates deeper learning due to its student-centered and active learning approaches (Grieve et al., 2017).

In terms of the existing teaching–learning practices of VC learning, FC (Flipped Classroom) with an element of virtual learning, González-Gómezet et al., (2016) found that when a direct comparison was made for students who followed the “flipped classroom” approach, performed better than the students taught in traditional methods. Despite having academic benefits, students also reported that they appreciated being able to learn when they wanted and they had the flexibility to review materials many times. González-Gómezet et al., (2016), found that students reported that the videos helped them achieve their learning goals for the class and, therefore, there is a need for videos to provide effective relevant material. González-Gómezet et al., (2016) also found that quizzes were of benefit to students to help direct students towards relevant content when learning virtually.

BL (Blended Learning) has been reported to help students engage more with the class as it was meant to be more interactive (Hande, 2014). Students in Indonesia commented that they liked opportunities to collaborate with BL (Mulyadi & Purnama, 2019). Students can easily communicate with teachers through forums and ICT (Hande, 2014) and students liked the mix of being able to use online platforms accommodated in BL (Mulyadi & Purnama, 2019). BL can also lend itself to a variety of learning styles and speeds of learning (Hande, 2014) and can help students identify gaps in their own knowledge (Hande, 2014) and, therefore, take more control over their learning. Mulyadi & Purnama, (2019) noted that students who use BL shared that social media assisted them with English learning (Mulyadi & Purnama, 2019). After receiving 985 valid questionnaires, López-Pérez et al., (2011) in a survey in Spain looking at BL found that fewer students dropped out of classes when BL was involved. Although BL is often focused on the use of ICT, this study by López-Pérez et al., (2011) found that BL increased motivation of students. Therefore, BL does not need to be seen as an opposition to face to face teaching but as complementary to it (López-Pérez et al., 2011).

VC learning is broadly divided into two categories: 100% online mode and mixed mode i.e., both online and offline options. Recent research has shown interest in both categories. For instance, according to Tang et al., (2020), despite students not being satisfied with online learning, those who took the blended learning course as opposed to synchronous 100% learning online had higher evaluations of the course as well as students having better attention. Blended learning has been shown to be an effective method to delivery teacher training during the pandemic (Rachmadtullah et al., 2020). Therefore, it can be adapted easily for any courses to many advantages. For instance, Law et al., (2022) in Hong Kong examined the students’ and teachers’ perceptions on online learning compared to face-to-face learning during Covid-19 and observed that from the teachers’ perspective, online learning depended on technical devices and systems that created stress and influenced teaching quality. As a result, Dang et al., (2020) concluded that it is the best to combine the face-to-face teaching with the positive aspects of online learning.

## VC learning in UAE

Within the UAE, platforms such as Microsoft teams, Zoom, Skype and Hangouts meet were all seen to be popular and used within the country for virtual learning (Al-Karaki et al., 2021). In a study, Masoud and Bohra (2020) concluded that the UAE government equipped teachers at government schools with specialist training to enhance virtual learning during the pandemic. Therefore, it was seen that, even from governmental level, the UAE was prioritizing the education of the students in the country to ensure the least impact possible during the Covid 19 pandemic.

A vital aspect of virtual learning is the teachers and instructors who prepare and deliver the online learning materials. Marín-Díaz et al., (2021) noted that students viewed the instructor’s role as one primarily of guidance and evaluation of completed work. Tang et al., (2020) also found that students placed communication with the teacher as highly important and were dissatisfied with virtual learning when there was poor communication. A study in Sharjah in the UAE, Guraya, (2020) saw how the College of Medicine at the University of Sharjah used technology to utilize an "e-flipped classroom" during the Covid 19 pandemic. Before attending a virtual class discussion led by an instructor, the student watched videos or recorded lectures to gain background knowledge on the topic. This was a novel way of accommodating the flipped classroom model during the Covid 19 pandemic to ensure the safety of all students involved. It also demonstrates the flexibility which is afforded by virtual learning to allow instructors to adapt materials and learning styles to the course being taught, as well as to the students.

The convenience to work at the time and place of the student choice is very valuable when undertaking virtual learning (Marín-Díaz et al., 2021). VC learning was seen by students as being very convenient (Hande, 2014). VC allows flexibility which students like (Mulyadi & Purnama, 2019). Almuraqab, (2020) also found that students in the UAE enjoyed the flexibility of virtual learning. Marín-Díaz et al., (2021) found that after surveying 431 students, that students did not use all the tools available on an online platform, with blogs, email and podcasts being the most popular forms of learning. There have even been differences seen in students’ perceptions of online learning related to location (urban or rural) and socioeconomic status, with Purushotham et al., (2020) finding both Urban students and those with higher income background felt more positive towards learning English through virtual learning compared with those in rural area with lower income backgrounds. Al-Maroof et al., (2021) found that students in the UAE were more likely to use e-learning if they felt more motivated towards this learning type. Self-efficacy also played a large role for students’ uptake of e-learning within the UAE. Salloum et al., (2019) undertook a study in Dubai during pre-Covid period, to look at factors which affected student acceptance of e-learning. They found that in the UAE, those students who were keen to seek knowledge, were keen to accept virtual learning. In addition, Salloum et al., (2019) found that the system used for the virtual learning must be of good quality and simple to use to encourage students to accept virtual learning.

## Teacher-student interaction of VC learning

Virtual learning platforms, when set up and optimized, can provide good collaboration between instructors and students (Marín-Díaz et al., 2021). Whilst students have an important part to play within the virtual classroom, there is an important role for instructors and teachers too. In addition to this, how the course is developed and constructed needs to be taken into consideration as this will be slightly different in a virtual classroom than for to face to face class.

Phillips and O'Flaherty, (2019) studied the concept of the virtual flipped classroom amongst university students. Although there was not significant difference found in student's academic performance when comparing the virtual flipped classroom to the traditional method of teaching for these nursing students, they did find that the course tutors played a large role. Students did not have a positive tutor experience with the virtual flipped classroom approach and found that tutors were often underprepared and lacked training on how to use the virtual classroom effectively. Therefore, Phillips and O'Flaherty, (2019) advocated for more professional development for those staff who use the VC to maximise effectiveness for the students.

Kavrayici, (2021) looked at the relationship between classroom management and the sense of classroom community in a graduate setting in Turkey. He found that when students perceived that the leadership of the course planned lessons suitable for the virtual environment. Good communication as well as the factors mentioned above led to students having better learning outcomes for online virtual learning. Therefore, careful planning is needed when instructors are thinking about virtual classroom to allow good communication, as well as the course structure and its implementation which are put in place effectively to give students the best possible opportunities during the virtual classroom. The sense of community online is vital to keep students enrolled in the virtual classroom as well as to promote collaboration within the virtual environment (Kavrayici, 2021). Therefore, it is to benefit the student and the institution. Learning communities are set up in virtual learning environments to encourage students to learn pro-actively and maintain retention of student numbers on online virtual learning courses.

## Advantages and drawbacks of VC learning

Like any other technology supported teaching–learning approaches, VC learning has several advantages. First, students learning autonomy is one of the most important benefits that VC learning can provide. For instance, in a Burnett (2011) study, one student noted how she liked to go online to use VC learning for 20 min per day and this enabled her to stay on the top of the course and aware of what was coming up. Similarly, Francecucci and Foster (2013) conducted a study comparing a virtual interactive and real-time instruction-led classroom and found that students who used the virtual classroom had significantly more interest in the course than those who did not use the virtual classroom. In Pakistan, Perveen, (2016) found that after looking at the responses from 1025 surveys, that a synchronous mode of learning English was preferred by the majority (82%) of students. Perveen, (2016) suggests that this was because of the student’s preference for face-to-face learning.

On the other hand, there are many factors which need to be coordinated in order for VC to be effective, so it can be a logistical task (Hande, 2014). Almuraqab, (2020) conducted a survey in Dubai to establish student's views on distance learning in the UAE though the Covid 19 pandemic. More than half of those surveyed liked distance learning, however only 49% of students would like to return to blended learning in the future. 60% of students identified that they had difficulty in establishing a firm understanding of their learning due to the virtual learning and physical distance from the instructor, with almost 58% finding group collaboration difficult online.

Another weakness is that it relies on good internet connectivity and suitable devices (Anastasakis et al., 2021; Marín-Díaz et al., 2021). The flexibility which comes with virtual learning is one hugely positive factor but can contribute to many of the other issues such as difficulty in communication and lack of connection with instructors (Swanson et al., 2021) Tang et al., (2020) found that when students came up against difficult topics, more traditional teaching methods were better than online teaching. Similarly, in a study of Hong Kong tertiary educational institutions, Law et al., (2022) concluded that teacher-student and student–teacher interactions were the biggest challenge in online learning that affected the acquisition and application of knowledge. The lack of hands-on experience was seen to be a drawback of virtual learning in the UAE (Al-Karaki et al., 2021) In a study by Swanson et al., (2021) looking at student’s experiences of the move from face to-face to distance learning in two universities in the United States, found that many students required more input, feedback and assurance from professors on the course. Technology failures and lack of interpersonal relations between students were also seen as leading to increased dissatisfaction with virtual learning. Swanson et al., (2021) advocate the provision of clear expectations and information to students who undertake virtual learning could mitigate many of the problems associated with virtual learning.

## Aims and research questions

The current research pursues to enhance understanding of students’ experience and of EdTech supported teaching and learning in general and in relation to VC learning, in particular. Perception of VC in this study refers to how students evaluate VC as an active learning practice; what kind of teacher-student interactions in VC classes; how they perceive its role in participatory learning approach; how they take position on their choice to take in VC classes and its drawbacks. Research perceptions of VC learning are significant because there are limited and inconclusive evidence about its effectiveness.

Although existing research has a major focus on experimentation and on stakeholder perceptions of online learning in various contexts in the globe (Burnett, 2011; Kavrayici, 2021; Perveen, 2016; Tang et. al., 2020), there has been limited research on its perceptions among university level students and other stakeholders in Gulf countries like the UAE. In this article, various perceptions of VC learning in relation to critical factors including students’ motivation of using ICT in VC classes and geographic location have been explored. This study focuses on the following research questions:

- To what extent do university students in the UAE perceive VC learning experiences in terms of its need and their choice to take up VC classes?
- What do teacher-students interact in classes and its advantages and drawbacks due to EdTech skills?
- To what extent do teachers and students organize study and homework time in VC classes?

The research on VC learning presently available advocates strong positive perceptions of the role of VC learning, regardless of its effectiveness (Burnett, 2011; Francecucci & Foster, 2013; Perveen, 2016; Elshami et al., 2021; farkhani et al., 2022). Although participation in the online learning is mediated by socioeconomic and locational factors (Purushotham et al., 2020), there has been limited study on whether differences in the perceptions of VC learning are related to these variables.

## Research design

**Fig. 1:**
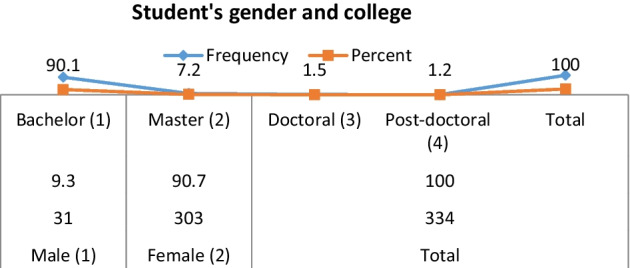
Student's gender and college

**Fig. 2:**
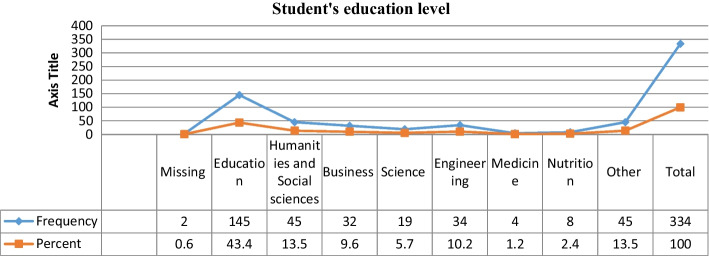
Student's education level

The main tool for data collection was questionnaire survey on VC learning in the UAE with the aid of audio-records in the present study. The questionnaire was circulated to 458 university learners in a range of disciplines from under graduation to postdoctoral stage and 334 participated in the survey. To ensure diversity in participants, students from a range of faculties were chosen. Even though the population of study was not on a national scale representative, participants were varied in terms of course, institution of higher education and class. Support was required from appropriate heads of the department in choosing the sample of learners from their student population (Figs. 1, 2).

Out of 334 students, 9.3% were male and 90.7% were female. Most of the participants are enrolled in the bachelor level degree (90.1%), while some are in the master level (7.2%), doctoral level (1.5%), or post-doctoral level (1.2%).

They above chart represents learners who have different specializations. The majority of them are from the College of Education (43.4%), followed by Humanities and Social sciences (13.5%), Engineering (10.2%), Business (9.6%), Science (5.7%), Nutrition (2.4%), Medicine (1.2%), and other colleges (13.5%). All the participants are from UAE.

The learner questionnaire was designed drawing from the previous research on the role of VC classes and face-to-face traditional instruction aspects in teaching–learning experiences and results in diverse contexts. The 27-item questionnaire was divided into 2 sections including gender, education level and students’ college. Other segment collected information on various aspects of students’ perception and experiences in VC classes including teaching, learning, teacher-student interaction, and the use of ICT, learners’ perspective about VC, feedback, motivation, and support.

### Findings

The section presents the findings by addressing the research questions.

### Students experience and choice to take up VC learning

We explore students’ perceptions and experiences of VC learning in relation to three topics choice to take up VC learning, teacher-students interact and homework time during learning. The survey data show that 82% of the students together agreed and strongly agreed positively for their experiences of flexibility in VC learning during the Covid-19 pandemic. Some key experiences of VC learning, as presented in Table 1, include the following:

- About78% of students together agreed and strongly agreed that they had learnt things that were more relevant to their life and interests while about 18% were neutral.
- About 79% of students together agreed and strongly agreed that VC learning had supported them to learn more independently while about 16% were neutral.
- About 72% of participants together agreed and strongly agreed that teachers had shown more trust in students in VC learning.
- About 79% of students together agreed and strongly agreed that they had worked hard and felt motivated to learn more while about 17% were neutral.
- About 76% of students together agreed and strongly agreed that they had achieved better in VC learning while 18% were neutral.
- 77% of students together agreed and strongly agreed that they had learned more study skills that would be useful after leaving university.
- About 76% of students together agreed and strongly agreed that their teachers had been better prepared and equipped for the lessons while about 18% were neutral.
- However, about 72% of students together agreed and strongly agreed that they had less interaction with other students.

**Table 1:** Learning experiences in VC learning

Items	Strongly agree		Agree		Neutral		Disagree		Strongly disagree		Mean	SD
	Freq	%	Freq	%	Freq	%	Freq	%	Freq	%		
I feel flexibility in VC	95	28.4	17.9	53.6	57	17.1	2	0.6	1	0.3	4.09	0.706
I learn things that are relevant to my life and interests	10	29.9	16	47.9	61	18.3	10	3	3	0.9	4.03	0.827
I learn more independently	85	25.4	18.2	54.5	54	16.2	11	3.3	2	0.6	4.01	0.777
My teacher shows more trust in students	91	27.2	16.8	50.3	63	18.9	10	3	2	0.6	4.01	0.798
I work harder and feel more motivated	83	24.9	18.1	54.2	59	17.7	6	1.8	5	1.5	3.99	0.796
I have better achievement	88	26.3	17.2	51.5	58	17.4	13	3.9	3	0.9	3.99	0.822
I learn study skills that will be useful after I leave university	91	27.2	16.7	50	54	16.2	18	3.4	4	1.2	3.97	0.871
My teacher is better prepared and equipped for the lesson	82	24.6	17.5	52.4	63	18.9	12	3.6	2	0.6	3.97	0.795
I have less interaction with other students	84	25.1	15.9	47.6	77	23.1	11	3.3	3	0.9	3.93	0.832

**Fig. 3:**
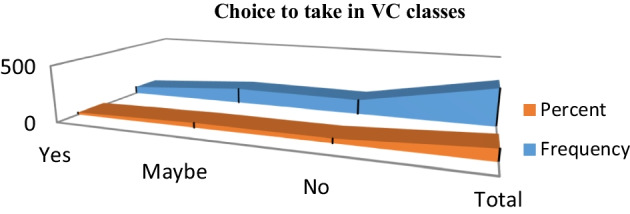
Choice to take up VC learning

The finding shown in (Fig. 3) indicate that the participants answered the question related to their choice to take in VC learning again: Yes (20.1%), no (38.3%), or maybe (41.6%).

**Fig. 4:**
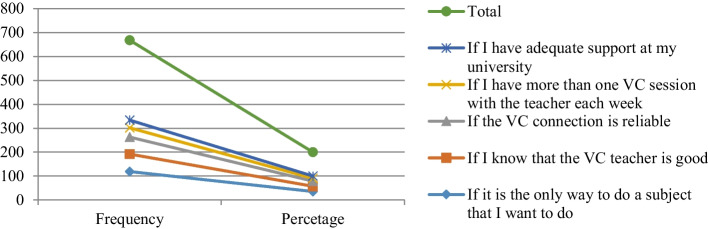
Factors that motivate learning through VC learning

The finding shown in (Fig. 4) indicate that the participants’ answers related to factors that motivate learning through VC learning, the highest percentage was if it is the only way to do a subject that they want to do (35.6%), if they know that the VC teacher is good (21.9%), if the VC connection is reliable (21.3%). Lower responses were given to the following items: If they have more than one VC session with the teacher each week (11.7%), and if they have adequate support at my university (9.6%).

### Teacher-student interaction in VC learning

The Table 2 shows the results of the survey on teacher-student interaction of VC learning. the results demonstrated that almost half of the participants (55.4%) indicated that the teacher talks most of the session throughout most or all of the classes. While (38.9%) indicated that this happens in some classes. Similarly, half of the participants (55.1%) indicated that the teacher shows the class multimedia presentations (e.g., PowerPoint, videos) in most or all of the classes. While (38.9%) indicated that this happens in some classes. The participants thought that students ask the teacher questions in most or all of the classes (44.6%) or in some classes (44.3%). Only (9%) of them thought that this happens in few classes. In addition, half of the participants (51.8%) indicated that the teacher goes through their assignments / homework in some classes. While (38.9%) indicated that this happens in most or all classes. Only (8.4%) of them thought that this happens in few classes. The participants thought that the teacher facilitates discussion between students in most or all of the classes (40.7%) or in some classes (46.1%). Only (11.7%) of them thought that this happens in few classes. They also indicated that some students and teachers experience technical difficulties (e.g. losing visual/audio) that disrupt teaching in some classes (44.0%), or in most/all classes (38.6%). Only (15.6%) of them thought that this happens in few classes. Finally, they indicated the teacher directs questions for individual students in some classes (42.5%), or in most/all classes (36.2%). Only (16.5%) of them thought that this happens in.

**Table 2:** Teacher-students interactions in VC learning

Items	None of the class		A few classes		Some classes		Most/All classes		Mean	SD
	Freq	%	Freq	%	Freq	%	Freq	%		
The teacher talks throughout most of the session	22	0.6	17	5.1	130	38.9	185	55.4	3.49	0.634
The teacher shows the class multimedia presentations	22	0.6	18	5.4	130	38.9	184	55.1	3.49	0.628
Students ask the teacher questions	77	2.1	30	9	148	44.3	149	44.6	3.31	0.723
The teacher goes through our assignments/homework	33	0.9	28	8.4	173	51.8	130	38.9	3.29	0.654
The teacher facilitates discussion between students	35	1.5	39	11.7	154	46.1	136	40.7	3.26	0.719
technical difficulties that disrupt the class	66	1.8	52	15.6	147	44	129	38.6	3.19	0.76
The teacher directs questions for individual students	116	4.8	55	16.5	142	42.5	121	36.2	3.1	0.843

The finding shown in (Table 3) indicate that the participants access to VC platform (e.g., blackboard) to download homework, post discussions, etc. either 3:4 times a week (38%) or 5 or more times a week (36.5%). While (23.4%) said that they do this 1:2 times a week. They indicated that they discuss their work with VC students within their university either 3:4 times a week (42.8%) or 5 or more times a week (31.7%). While (19.5%) said that they do this 1:2 times a week. In addition, they search the internet for useful websites for their VC class either 3:4 times a week (45.2%) or 5 or more times a week (29.9%). While (19.2%) said that they do this 1:2 times a week. The participants indicated that they use study time to work on their VC assignments/homework either 3:4 times a week (43.4%) or 5 or more times a week (27.2%). While (25.1%) said that they do this 1:2 times a week. They also indicated that they access other websites and videos recommended by their VC teacher 3:4 times a week (40.4%) or 5 or more times a week (28.4%). While (25.7%) said that they do this 1:2 times a week. The participants’ answers regarding how many time of them mail/text/call their VC teacher for help was as follows: 3:4 times a week (36.6%), 1:2 times a week (31.7%), and 5 or more times a week (25.7%). They indicated that they approach teacher(s) within their university who teach/know the subject for help: 3:4 times a week (38.6%), 1:2 times a week (24.3%), and 5 or more times a week (27.2%). Finally, the participants indicated that they have one-on-one VC sessions with their VC teacher: 3:4 times a week (38.6%), 1:2 times a week (23.4%), and 5 or more times a week (22.5%). Only (15.6%) indicated that they rarely or never do this.

**Table 3:** study/homework time in VC learning

Items	Rarely/Never		1:2 times a week		3:4 times a week		5 or more times a week		Mean	SD
	Freq	%	Freq	%	Freq	%	Freq	%		
I access my VC platform to download homework	7	2.1	78	23.4	127	38	122	36.5	3.09	0.82
I discuss my work with other VC students	20	6	65	19.5	143	42.8	106	31.7	3	0.87
I search the Internet for useful websites for VC class	19	5.7	64	19.2	151	45.2	100	29.9	2.99	0.85
I use study time to work on assignments/homework	14	4.2	84	25.1	145	43.4	91	27.2	2.94	0.83
I access other websites and videos recommended by my VC teacher	18	5.4	86	25.7	135	40.4	95	28.4	2.92	0.87
I mail/text/call my VC teacher for help	19	5.7	106	31.7	123	36.6	86	25.7	2.83	0.88
I approach teacher within my university who teach the subject for help	33	9.9	81	24.3	129	38.6	91	27.2	2.83	0.94
I have one-on-one VC sessions with my VC teacher	52	15.6	78	23.4	129	38.6	75	22.5	2.68	0.99

## Discussion and conclusion

VC learning has become popular as an EdTech instruction tool during COVID-19 situation. In a study in China, Zhang (2019) concludes that students have shown interest in enrolling in VC learning that needs to be acknowledged. We surveyed 334 university students who used VC learning to understand their perceptions and experiences. Overall, the findings demonstrated that VC learning approaches proved necessary among students. The overall experiences of taking up VC learning were significantly positive in response to teacher-student interaction and doing the study and homework properly. These findings confirm an earlier study by Masoud and Bohra (2020), which concludes that VC learning increases access to education in the COVID-19 era and beyond. The results also endorse a previous study by Mulyadi & Purnama (2019) in Indonesia that concludes students' satisfaction with online or blended learning environment. So, students’ experiences of VC learning suggest that although they are forced to take up VC learning due to the Covid19 crisis, they become aware of its necessity which motivates them to continue their studies using EdTech pedagogies. In another study in Indonesia, Aditya et al. (2019) found the benefits of VC learning with many challenges and difficulties. This study showed significant consequences as a pointer to the implementation of VC learning approaches for vocational students in Indonesia. In terms of teacher-student interaction in VC learning, Willermark (2021) conducted a study to understand the different aspects of interaction in VC learning pedagogy in Sweden and drew multiple pictures of interaction that involved both increased and reduced contact with the students and their activities. The study also concluded the complex role of interaction in VC learning and provided implications for teachers and students.

Although the data indicates the positive experiences of VC learning, students classify the importance of the teacher-student relationship. Most of the students agree that teachers’ role is crucial when they conduct VC learning. This echoes other studies which highlighted the vital role of the teacher in VC learning (Almusharraf, 2021; Tang et al., 2020). If teachers talk less and do not communicate with students properly, VC learning becomes less attractive which raises the question of the efficacy of VC learning approaches. In a large-scale study in china, Tang et al., (2020) resolved those students were not satisfied with online learning in general, and they were particularly not satisfied with the communication when teaching-leaning continued with 100% online. However, they felt positive and identified that if classes continued using blended or flipped learning modes that improved their learning, attention and evaluation.

In terms of doing the homework/assignment using the VC platform, the data indicate a significantly positive role (78%) as the majority of students download the homework/assignment at least 1 or 2 times a week and they discuss their work among their peers. Students also show their encouraging attitudes (84%) toward using other EdTech skills as per the recommendation of their VC class teachers which categorically proves how students motivate to continue their study using the VC platform in the Covid-19 crisis. However, students were less interested to take up one-on-one VC sessions with their VC teacher. Finally, the UAE university students indicate their inspiring motivation to use ICT for VC classes. Most students use the internet independently and search the necessary website for their studies.

It is clear to be seen that the virtual classroom has many advantages which can be drawn upon in the time of the COVID-19 pandemic allowing higher education to continue and ensuring uninterrupted learning for those students enrolled in university courses. Gallagher (2020) reveals the acceptance of high-quality education technology and digital capabilities across thousands of colleges and universities. Denotes that the year marks a clear inflexion point as students, educators and government leaders alike analyze the price and value proposition of higher education through the new lens of the traditional classroom versus multiple modes of digital delivery. The implementation of distance education through online mode which was in demand during the pandemic period served its purpose worldwide and this has been proved in one of the statistical surveys conducted in the Southeast University in China, among 39,854students during the Pandemic quarantine. Most of the students agreed that the virtual classroom enabled the continuity of their education and it also helped them to escape the distress due to quarantine as the teachers were able to bring the classroom spirit to their residing places through online platforms. The virtual mode also served as a meter to test the students in terms of self-discipline, interest and focus in the class and tolerance and remedy towards the hindrance caused (Sun, et al., 2020). Haider and Al-Salman (2020) did an analysis at Jordanian University to study the psychosomatic impact of the Covid-19 e-learning platform and they stated that the respective method will help students to develop their skills in them with a futuristic vision.

United Arab Emirates continues to strive to provide a world-class education system within a global education market. Therefore, it is vital that new technologies are implemented to provide seamless education to those students within the UAE during this Covid pandemic time. The virtual classroom has been shown to afford creativity and provide a flexible learning environment for many students in higher education. It can play a part in increasing achievement as well as engaging students. However, there is a need for learning communities within the virtual classroom situation to allow students to interact and learn from one another. Instructors and those who design virtual classroom courses need to lead and design these modules to maximise the use of the virtual classroom and minimise any disadvantages such as lack of face-to-face communication.

## Data Availability

The datasets used and/or analysed during the current study are available from the corresponding author on reasonable request.
