# Correction: UAE university students’ experiences of virtual classroom learning during Covid 19

**DOI:** 10.1186/s40561-023-00232-2

**Published:** 2023-02-07

**Authors:** Monjurul Islam, Nurul Hijja Mazlan, Ghadah Al Murshidi, Mohammed Shamsul Hoque, S. V. Karthiga, Mohoshin Reza

**Affiliations:** 1 School of Language, Literacies and Translation, Universiti Sains Malaysia, Penang, Malaysia; 2 College of Computing, Informatics, and Media, Universiti Teknologi MARA, Shah Alam, Malaysia; 3 United Arab Emirates University, College of Education, Al Ain, UAE; 4 Department of English, Daffodil International University, Dhaka, Bangladesh; 5 College of Science and Humanities, SRM Institute of Science and Technology, Kattankulathur, India; 6 Department of English, Bangladesh University of Professionals, Dhaka, Bangladesh

**Correction: Smart Learning Environments (2023) 10:5** https://doi.org/10.1186/s40561-023-00225-1

Following publication of the original article [1], the authors reported that the affiliations were missing in the submitted manuscript and were not present in the published article. The correct affiliations are presented in this correction article and the original article [1] has been updated.
